# Use of inpatient palliative care in metastatic testicular cancer patients undergoing critical care therapy: insights from the national inpatient sample

**DOI:** 10.1038/s41598-024-83545-7

**Published:** 2025-01-06

**Authors:** Cristina Cano Garcia, Reha-Baris Incesu, Francesco Barletta, Simone Morra, Lukas Scheipner, Andrea Baudo, Stefano Tappero, Mattia Luca Piccinelli, Zhe Tian, Fred Saad, Shahrokh F. Shariat, Carlo Terrone, Ottavio De Cobelli, Luca Carmignani, Sascha Ahyai, Nicola Longo, Derya Tilki, Alberto Briganti, Severine Banek, Luis A. Kluth, Felix K. H. Chun, Pierre I. Karakiewicz

**Affiliations:** 1 Cancer Prognostics and Health Outcomes Unit, Division of Urology, University of Montréal Health Center, Montréal, QC Canada; 2 Department of Urology, Goethe University Frankfurt, University Hospital Frankfurt, Theodor-Stern-Kai 7, Frankfurt, Germany; 3 Martini-Klinik Prostate Cancer Center, University Hospital Hamburg-Eppendorf, Hamburg, Germany; 4 Unit of Urology/Division of Oncology, Gianfranco Soldera Prostate Cancer Lab, IRCCS San Raffaele Scientific Institute, Milan, Italy; 5 Vita-Salute San Raffaele University, Milan, Italy; 6 Reproductive Sciences and Odontostomatology, Urology Unit, Department of Neurosciences, University of Naples Federico II, Naples, Italy; 7 Department of Urology, Medical University of Graz, Graz, Austria; 8 Department of Urology, IRCCS Ospedale Galeazzi – Sant’Ambrogio, Milan, Italy; 9 Department of Urology, IRCCS Policlinico San Donato, Milan, Italy; 10 Department of Urology, Ospedale Policlinico San Martino, Genoa, Italy; 11 Department of Surgical and Diagnostic Integrated Sciences (DISC), University of Genova, Genoa, Italy; 12 Department of Urology, IEO European Institute of Oncology, IRCCS, Milan, Italy; 13 Department of Urology, Comprehensive Cancer Center, Medical University of Vienna, Vienna, Austria; 14 Department of Urology, Weill Cornell Medical College, New York, NY USA; 15 Department of Urology, University of Texas Southwestern, Dallas, TX USA; 16 Hourani Center of Applied Scientific Research, Al-Ahliyya Amman University, Amman, Jordan; 17 Department of Urology, University Hospital Hamburg-Eppendorf, Hamburg, Germany; 18 Department of Urology, Koc University Hospital, Istanbul, Turkey

**Keywords:** Inpatient palliative care, End-of-life care, Metastatic testicular cancer, Critical care therapy, NIS, Germ cell tumours, Testicular cancer, Epidemiology

## Abstract

To test for rates of inpatient palliative care (IPC) in metastatic testicular cancer patients receiving critical care therapy (CCT). Within the Nationwide Inpatient Sample (NIS) database (2008–2019), we tabulated IPC rates in metastatic testicular cancer patients receiving CCT, namely invasive mechanical ventilation (IMV), percutaneous endoscopic gastrostomy tube (PEG), dialysis for acute kidney failure (AKF), total parenteral nutrition (TPN) or tracheostomy. Univariable and multivariable logistic regression models addressing IPC were fitted. Of 420 metastatic testicular cancer patients undergoing CCT, 70 (17%) received IPC. Between 2008 and 2019, the rates of IPC among metastatic testicular cancer patients undergoing CCT increased from 5 to 19%, with the highest rate of 30% in 2018 (EAPC: + 9.5%; 95% CI + 4.7 to + 15.2%; p = 0.005). IPC patients were older (35 vs. 31 years, p = 0.01), more frequently had do not resuscitate (DNR) status (34 vs. 4%, p < 0.001), more frequently exhibited brain metastases (29 vs. 17%, p = 0.03), were more frequently treated with IMV (76 vs. 53%, p < 0.001) and exhibited higher rate of inpatient mortality (74 vs. 29%, p < 0.001). In multivariable analyses, DNR status (OR 10.23, p < 0.001) and African American race/ethnicity (OR 4.69, p = 0.003) were identified as independent predictors of higher IPC use. We observed a significant increase in rates of IPC use in metastatic testicular cancer patients receiving CCT, rising from 5 to 19% between 2008 and 2019. However, this rates remain lower compared to metastatic lung cancer patients, indicating the need for further awareness among clinicians treating metastatic testicular cancer. The increase in IPC rates for metastatic testicular cancer patients receiving CCT indicates a need for ongoing education and awareness among healthcare providers. This could enhance the integration of IPC in the treatment of advanced cancer, potentially improving quality of life and care outcomes for survivors.

## Introduction

The integration of inpatient palliative care (IPC) represents a well-established recommendation for advanced cancer patients^1–3^. In general, elevated rates of IPC are considered a favorable quality-of-care indicator^4–6^. For example, breast and lung cancer patients receiving critical care therapy (CCT) that represent landmark cancer diseases regarding palliative care, described IPC rates of 22% and 28%, respectively^7^. However, the rate of IPC in metastatic testicular cancer patients receiving CCT is unknown. We addressed this knowledge gap and tested for IPC rates in metastatic testicular cancer patients who received CCT, namely invasive mechanical ventilation (IMV), percutaneous endoscopic gastrostomy tube (PEG), dialysis for acute kidney failure (AKF), total parenteral nutrition (PEN) or tracheostomy, within the Nationwide Inpatient Sample (NIS) 2008–2019, according to previously used methodology^7–9^. We hypothesized that rates of IPC are comparable to those recorded in metastatic lung and breast cancer patients.

## Methods

### Data source

We relied on the NIS database (2008–2019) to assess trends in and predictors of IPC use in patients receiving CCT. The NIS represents a set of longitudinal hospital inpatient databases included in the Healthcare Cost and Utilization Project (HCUP), created by the Agency for Healthcare Research and Quality (AHRQ) through a federal-state partnership. The database includes approximately 20% of the United States inpatient hospitalizations^10^. It uses stratified sampling to ensure the sample reflects the diversity of hospitals in terms of geographic region, size, urban vs. rural location and teaching status. All diagnoses were coded using the International Classification of Diseases (ICD)-9-Clinical Modification (ICD-9-CM) and ICD-10-CM, as well as all procedures were coded using ICD-9-Procedure Codes (PCS) and ICD-10-PCS.

### Study population

We focused on patients aged ≥ 18 years with a diagnosis of metastatic testicular cancer (ICD-9-CM code 186.9, ICD-10-CM codes C62.90, C62.92, C62.91, C62.10, C62.11, C62.12), who received CCT. Specifically, CCT included IMV (ICD-9-PCS codes 96.70–96.72, ICD-10-PCS codes (5A1.935Z, 5A1.945Z, 5A1.955Z), PEG (ICD-9-PCS code 43.11, ICD-10-PCS codes 0DH.63UZ, 0DH.64UZ), dialysis for AKF (ICD-9-CM codes 584.5, 584.6, 584.7, 584.8, 584.9 and ICD-9-PCS 39.95, ICD-10-CM N17.0, N17.1, N17.2, N17.8, N17.9 and ICD-10-PCS 5A1.D70Z, 5A1.D80Z, 5A1.D90Z), TPN (ICD-9-PCS code 99.15, ICD-10-PCS 3E0.336Z, 3E0.436Z, 3E0.536Z, 3E0.636Z), and tracheostomy (ICD-9-PCS codes 31.1, 31.21, 31.29, ICD-PCS codes 0B1.10F4, 0B1.10Z4, 0B1.13F4, 0B1.13Z4, 0B1.14F4, 0B1.14Z4).

### Statistical analysis

We evaluated IPC rates and inpatient mortality in metastatic testicular cancer patients receiving CCT. Estimated annual percentage changes (EAPC) were tested with the least squares linear regression. Moreover, univariable and multivariable logistic regression models tested for predictors of IPC use. Adjustment variables consisted of age at admission, race/ethnicity (Non-Hispanic Whites vs. Hispanics vs. African Americans vs. others), do not resuscitate (DNR) status, presence of brain metastases, IMV treatment and TPN administration. We used survey-specific methods to account for the sampling design of the NIS, incorporating discharge weights to ensure that the results were nationally representative and using generalized estimating equations discharge to account for hospital clusters.

In all statistical analyses, R software environment for statistical computing and graphics (R version 4.1.3, R Foundation for Statical Computing, Vienna Austria) was used^11^. All tests were two sided, with a significance level set at p < 0.05. Owing to the anonymously coded design of the NIS, study-specific ethics approval was waived by the institutional review board.

## Results

### Descriptive characteristics

Relying on the NIS (2008–2019), we identified 420 patients with metastatic testicular cancer treated with CCT. Specifically, 240 (57%) patients received IMV, 151 (36%) received TPN, 65 (15%) underwent dialysis for AKF, 19 (5%) required PEG placement and 14 (3%) required a tracheostomy. Of those, 70 (17%) received IPC. IPC patients were older (35 vs. 31 years, p = 0.01), more frequently had DNR status (34 vs. 4%, p < 0.001), more frequently exhibited brain metastases (29 vs. 17%, p = 0.03), were more frequently treated with IMV (75 vs. 54%, p < 0.001) and exhibited higher rate of inpatient mortality (74 vs. 29%, p < 0.001). Conversely, IPC patients less frequently received TPN (p < 0.001). No differences between IPC vs. no-IPC patients were observed regarding length of stay, race/ethnicity, teaching hospital status, insurance, income, large bed size, pulmonary metastases, liver metastases, primary diagnosis at admission and CCT other than IMV and TPN (Table 1).

**Table 1:** Baseline characteristics of 420 metastatic testicular cancer patients who received critical care therapy (CCT), stratified according to inpatient palliative care (IPC) vs. no IPC.

Characteristics		Overall (420)	IPC n = 70 (17%)^1^	no IPC n = 350 (83%)^1^	p-value^2^
Age at admission in years		32 (25, 44)	35 (28, 44)	31 (24, 43)	0.01
Length of stay in days		13 (7, 22)	11 (4, 20)	14 (7, 22)	0.05
Length of stay in days (only survivors n = 266)		15 (8, 24)	13 (8, 27)	16 (8, 24)	0.7
Race/ethnicity	Non-Hispanic Whites	244 (58%)	35 (50%)	209 (60%)	0.1
	Hispanics	96 (23%)	15 (22%)	81 (23%)	0.01
	African American	Black (5%)	10 (14%)	12 (3%)	
	Others^+^	58 (14%)	10 (14%)	48 (14%)	
Insurance	Private	182 (43%)	27 (39%)	155 (44%)	0.1
	Medicaid	145 (35%)	21 (30%)	124 (35%)	
	others	93 (22%)	22 (31%)	71 (20%)	
High income^++^		169 (40%)	23 (33%)	146 (42%)	0.2
Region	South	154 (37%)	27 (39%)	127 (36%)	0.6
	West	113 (27%)	20 (29%)	93 (27%)	
	Midwest	90 (21%)	*	*	
	Northeast	63 (15%)	*	*	
Teaching hospital		319 (76%)	53 (76%)	266 (76%)	> 0.9
Large bedsize^+++^		290 (69%)	54 (77%)	236 (67%)	0.1
Metastases	Pulmonary	190 (45%)	31 (44%)	159 (45%)	0.9
	Liver	113 (27%)	24 (34%)	89 (25%)	0.1
	Brain	81 (19%)	20 (29%)	61 (17%)	0.03
Primary diagnosis at admission	Cancer-related	223 (53%)	35 (50%)	188 (54%)	0.2
	Infection	63 (15%)	14 (20%)	49 (14%)	
	Cardiovascular/pulmonary	48 (11%)	37 (11%)	11 (16%)	
	other	86 (20%)	10 (14%)	76 (22%)	
Critical care therapy	IMV	240 (57%)	53 (76%)	187 (53%)	< 0.001
	Dialysis for AKF	65 (15%)	13 (19%)	52 (15%)	0.4
	TPN	151 (36%)	*	*	< 0.001
	PEG	19 (5%)	*	*	0.3
	Tracheostomy	14 (3%)	*	*	0.7
DNR		38 (9%)	24 (34%)	14 (4%)	
In-hospital death		154 (37%)	52 (74%)	102 (29%)	< 0.001

*IPC* inpatient palliative care, *IMV* invasive mechanical ventilation, *AKI* acute kidney failure, *TPN* total parenteral nutrition, *PEG* percutaneous endoscopic gastrostomy tube, *DNR* do not resuscitate.

^1^Median (IQR); n (%).

^2^Wilcoxon rank sum test; Pearson’s Chi-square test; Fisher’s exact test.

^+^Includes Asian or Pacific Islander, Native American, or other as coded on the Nationwide Inpatient Sample database.

^++^High income defined as third and fourth quartiles.

^+++^Large bedsize defined as ≥ 400.

*These values cannot be displayed due to the Nationwide Inpatient Sample database regulation.

### Annual trends of inpatient palliative care and inpatient mortality in metastatic testicular cancer patients receiving critical care therapy

**Fig. 1:**
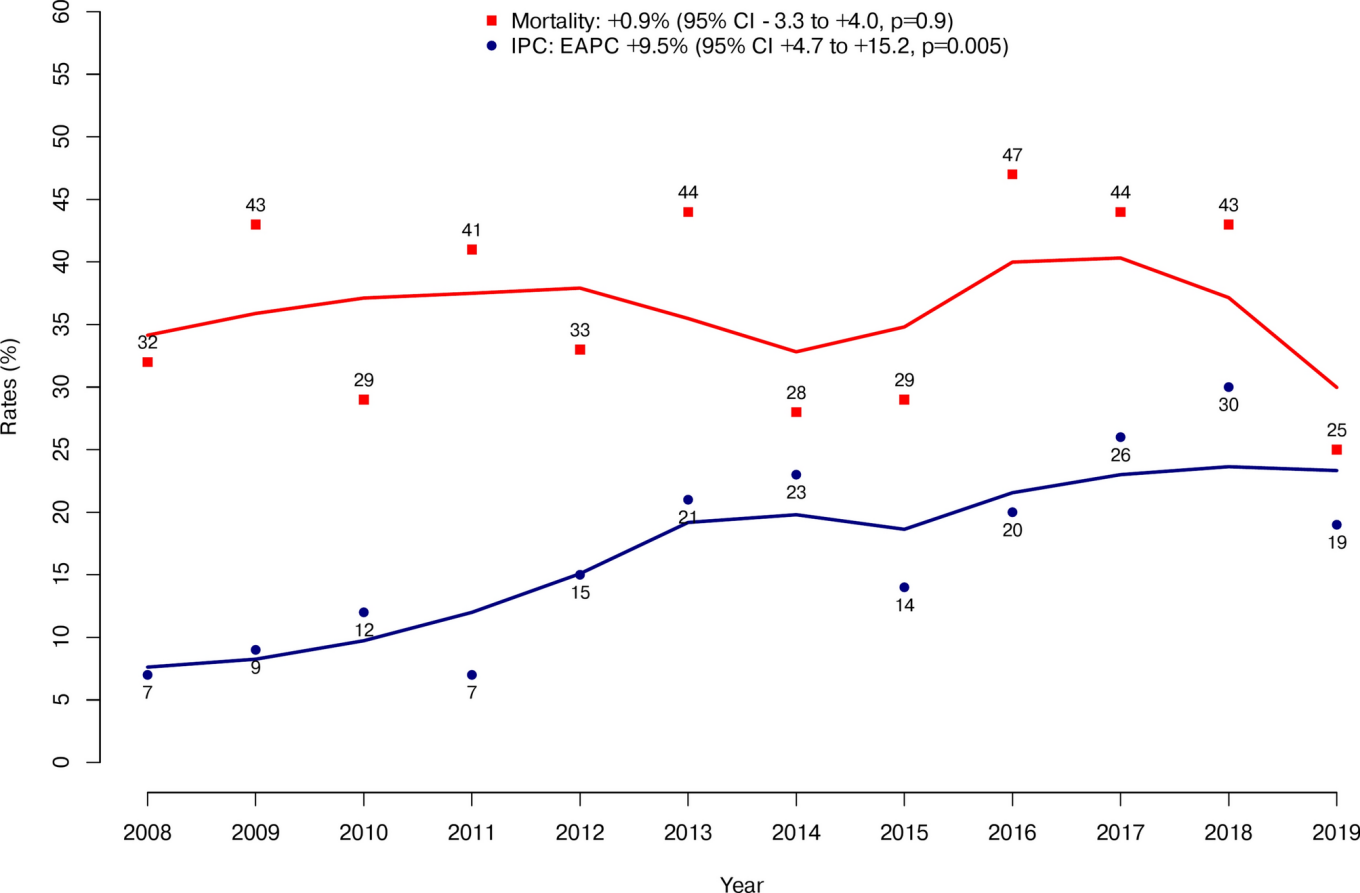
Rates of inpatient palliative care (IPC) in metastatic testicular cancer patients receiving critical care therapies (CCT) within the Nationwide Inpatient Sample (NIS) database from 2008 to 2019.

Between 2008 and 2019, the rates of IPC among metastatic testicular cancer patients undergoing CCT increased from 5 to 19%, with the highest rate of 30% in 2018 (EAPC: + 9.5%; 95% CI + 4.7 to + 15.2%; p = 0.005). However, between 2008 and 2019, the rates of mortality in metastatic testicular cancer patients did not increase significantly (EAPC + 0.9%, 95% CI − 3.3 to + 4.0%, p = 0.9, Fig. 1).

### Univariable and multivariable logistic regression models predicting inpatient palliative care in metastatic testicular cancer patients receiving critical care therapy

In univariable logistics regression models, older age (OR 1.02, 95% confidence interval [CI]) 1.00–1.04, p = 0.02), African American race/ethnicity (OR 4.87, 95% CI 1.99–11.91, p < 0.001), DNR status (OR 12.05, 95% CI 5.74–25.29, p < 0.001), presence of brain metastases (OR 1.79, 95% CI 1.01–3.15, p = 0.04) and IMV treatment (OR 2.49, 95% CI 1.41–4.39, p = 0.002) were associated with higher rate of IPC use. Conversely, TPN administration was associated with lower IPC use (OR 0.24, 95% CI 0.12–0.49, p < 0.001). In multivariable analyses, DNR status (OR 9.37, 95% CI 4.28–20.52, p < 0.001) and African American race/ethnicity (OR 4.69, 95% CI 1.65–13.35, p = 0.003) represented independent predictors of IPC use (Table 2).

**Table 2:** Univariable and multivariable logistic regression models predicting inpatient palliative care (IPC) in metastatic testicular cancer patients receiving critical care therapies (CCT).

Mortality	Univariable		Multivariable	
	OR (95% CI)	p-value	OR (95% CI)	p-value
Age at admission	1.02 (1.00–1.04)	0.02	1.01 (1.00–1.04)	0.2
Length of stay	0.98 (0.96–1.00)	0.10		
Race/ethnicity				
Non-Hispanic Whites	Reference	–	Reference	–
Hispanics	1.01 (0.53–1.94)	0.97	1.19 (0.58–2.45)	0.6
African American	4.87 (1.99–11.91)	< 0.001	4.69 (1.65–13.35)	0.003
Others	1.09 (0.50–2.38)	0.82	0.70 (0.25–2.00)	0.5
DNR	12.05 (5.74–25.29)	< 0.001	10.23 (4.60–22.71)	< 0.001
High income	0.75 (0.45–1.26)	0.2		
Insurance				
Private	Reference	–		
Medicaid	0.93 (0.52–1.66)	0.80		
Others	1.73 (0.94–3.17)	0.08		
Teaching hospital status	1.05 (0.57, 1.91)	0.88		
Large bedsize	1.81 (0.98–3.34)	0.06		
Pulmonary metastases	0.96 (0.58, 1.59)	0.88		
Liver metastases	1.48 (0.88–2.51)	0.14		
Brain metastases	1.79 (1.01–3.15)	0.04	1.62 (0.85–3.08)	0.1
Primary diagnosis at admission				
Others	Reference	–		
Cancer-related	1.39 (0.67–2.90)	0.38		
Cardiovascular/pulmonary	2.08 (0.83–5.24)	0.12		
Infection	2.04 (0.86–4.81)	0.10		
Critical care therapy				
IMV	2.49 (1.41–4.39)	0.002	1.12 (0.47–2.65)	0.8
Dialysis for AKF	1.28 (0.66–2.48)	0.47		
PEG	1.77 (0.60–5.25)	0.31		
TPN	0.24 (0.12–0.49)	< 0.001	0.37 (0.13–1.08)	0.07
Tracheotomy	1.30 (0.39–4.37)	0.7		

*OR* odds ratio, *CI* confidence interval, *IPC* inpatient palliative care, *IMV* invasive mechanical ventilation, *AKF* acute kidney failure, *TPN* total parenteral nutrition, *PEG* percutaneous endoscopic gastrostomy tube, *DNR* do not resuscitate.

## Discussion

We examined contemporary rates of IPC in metastatic testicular cancer patients receiving CCT within the NIS (2008–2019). We made several important observations.

First, we relied on previously reported methodology that identifies metastatic cancer patients receiving CCT as target population and in whom the rates of IPC represent an area of interest^7–9^. The current study cohort was substantially smaller (n = 420), due to the rarity of metastatic testicular cancer relative to metastatic bladder (n = 1944) or metastatic prostate cancer (n = 4168)^8,9^. Despite smaller sample size, the cohort of 420 metastatic testis cancer patients receiving CCT allowed analyses addressing IPC rates, IPC rates over time, as well as testing of associations between candidate predictors of IPC use. In consequence, population-based data repositories such as the NIS are essential to study rare events, such as metastatic testicular cancer with concurrent IPC use.

Second, within the cohort of interest of metastatic testicular cancer patients, who were treated with CCT, 70 (17%) received IPC. This rate is lower than the reference standard described in metastatic breast cancer (22%) or metastatic lung cancer (28%), where use of early palliative care is well-established^12–16^. In those disease models, early IPC has been shown to be associated with a number of favorable outcomes such as improved quality of life, lower depressive symptoms, lower length of stay, lower cost of hospitalization and lower readmission to acute care institutions^4–6,12,17^. However, the current IPC rate recorded in metastatic testicular cancer patients receiving CCT is higher than previously reported IPC rates in metastatic bladder (10%), as well as in metastatic prostate cancer (11%) patients treated with CCT. Nonetheless, the medical oncology community caring for metastatic testicular cancer patients should be further sensitized to the use of IPC with the intent of increasing this rate and ideally reaching the rates recorded in metastatic breast or metastatic lung cancer.

Third, although the overall rate of IPC in metastatic testicular cancer was lower than expected, relative to metastatic breast cancer and metastatic lung cancer, annual trends showed an increase from 5% in 2008 to 19% in 2019 with highest rate of 30% in 2018. In consequence, it may be postulated that most contemporary metastatic testicular cancer patients are benefitting from similar IPC rates, relative to their metastatic breast and lung cancer counterparts^7^. While this trend of increasing rate of IPC in metastatic testicular cancer is encouraging, increased awareness among clinicians treating patients with metastatic testicular cancer is needed. The young age of many metastatic testicular cancer patients makes them particularly vulnerable to psychological distress, which could be aggravated by CCT^18^. Therefore, it is concerning that only 17% of patients in this study received IPC. Although the rate of IPC is slightly higher among patients with brain metastases (25%, 61 out of 80), better selection of IPC for this group is still needed. Previous research has shown that patients with brain metastases, who typically have poor overall survival, are often treated with high-dose chemotherapy and multimodality therapies such as brain radiotherapy to manage symptoms that warrant an early integration of IPC^19^.

Fourth, we tested for differences in characteristics between IPC vs. no-IPC patients to identify predictors of IPC use.

We identified predictors of IPC use through univariable analysis, including older age (OR 1.02, p = 0.02), African American race (OR 4.87, p < 0.001), DNR status (OR 12.05, p < 0.001), brain metastases (OR 1.79, p = 0.04), and IMV treatment (OR 2.49, p = 0.002), while TPN administration was associated with lower IPC use (OR 0.24, p < 0.001). Multivariable analysis confirmed DNR status (OR 9.37, p < 0.001) as an independent predictor for IPC. This finding suggests that IPC is still seen mostly as end-of-life treatment as for the traditional approach of offering palliative care solely at the end of life. However, the contemporary approach is to introduce palliative care at diagnosis and gradually intensifies as the illness progresses^20^. Moreover, multivariable analysis confirmed African American race (OR 4.69, p = 0.003) as independent predictor for higher IPC use. It was previously described that African Americans exhibit the lowest survival rates in testicular cancer compared to other races/ethnicities, especially in patients with metastatic stage^21^. Conversely, in prostate cancer patients receiving CCT, African American race/ethnicity was associated with lower rates of IPC. These findings suggest that in metastatic testicular cancer cases requiring CCT, higher mortality rates lead to IPC being part of end-of-life care. In contrast, mortality rates in prostate cancer are lower, and IPC is more used as supportive care. This supportive care has previously been shown to be less frequently provided to African American patients^22^. Within the current analyses, available patient and tumor characteristics that were associated with higher IPC use in univariable analyses, could not independently predict IPC use in multivariable analyses, such as age, presence of brain metastases and IMV treatment. It should be noted that further key variables that were considered important in metastatic cancer patients, such as performance status, outpatient palliative referral, difference in laboratory values, symptom scores and opioid use are not available in the NIS database^14,23^. In consequence, these variables could not be included in the current analyses for purpose of formal statistical testing. However, absence of their considerations in the current univariable and/or multivariable analyses is not indicative of their lack of importance in the context of metastatic testicular cancer patients, in whom IPC should be considered.

Fifth, we observed higher inpatient mortality in IPC patients, relative to their no-IPC counterparts. These findings indicate that especially patients close to death are receiving IPC. This observation is in agreement with Loh et al. and Ruck et al., where similar observations have been made^7,24^. Under ideal conditions, IPC use should be associated with lower inpatient mortality. Specifically, expected deaths due to cancer progression should ideally not occur at acute care institutions, but instead after discharge from such facilities or institutions like hospices^25^. Jatwani et al. presented an abstract at ASCO Genitourinary Cancers Symposium 2024 about trends in hospice care utilization in testicular cancer in the United States of America from 2003 to 2020. The authors report a notable lower use of hospice services in testicular cancer patients aged 15–34 years compared to 55–74 years^26^. These findings support the need for earlier discussions about palliative care and if needed also in hospice care for younger patients.

Despite its novelty, the current study has several limitations. First and foremost, we relied on a previously established methodology that identifies CCT patients as targeted population where IPC rates represent an area of interest. Such approach is well-established^7–9^. Additionally, the NIS definition of IPC is based on ICD-9 and ICD-10 codes^10^. However, other methodological approaches for identification of IPC patients, as well as for IPC definition are also possible. In consequence, use of such approaches will result in IPC rates that may not be directly comparable. Second, rates of IPC require considerations in the context of clinical decision-making dynamics within each specific primary cancer patient group. For example, greater familiarity with the treated natural history of metastatic lung cancer may result in broader use of IPC than in other more rare primaries. Metastatic testicular cancer clearly qualifies for the status of a more rare primary where the treated natural history is less established. In consequence, although IPC represents an overall established indicator of favorable quality of care in metastatic cancer patients, absolute IPC rates are not directly comparable between different primaries. Third, we relied on a large-scale retrospective database with its inherent limitations including both selection and reporting biases. Fourth, our analyses relied on limited number of events. In consequence, ideally even larger scale databases than NIS could potentially provide more robust results. Finally, the NIS exclusively provides inpatient palliative care and inpatient mortality data. However, palliative care may be delivered after discharge^2,4,12,27^. In consequence, inpatient data may underestimate overall palliative care rates that address inpatient and outpatient care.

## Conclusions

Between 2008 and 2019, the rates of IPC among metastatic testicular cancer patients undergoing CCT increased significantly from 5 to 19%, with the highest rate of 30% in 2018. However, this rate remains lower compared to metastatic lung cancer patients, indicating the need for further awareness among clinicians treating metastatic testicular cancer.

## Data Availability

The datasets generated during and/or analyzed during the current study are available in the National (Nationwide) Inpatient Sample (NIS) repository (https://hcup-us.ahrq.gov/tech_assist/centdist.jsp).
